# Should temporary hemodialysis catheter insertion remain a requirement of nephrology residency training?

**DOI:** 10.1186/s40697-015-0042-0

**Published:** 2015-03-05

**Authors:** Arsh K Jain

**Affiliations:** London Health Sciences Centre, 800 Commissioners Rd. E., Room ELL-101, London, ON N6A 4G5 Canada

## Abstract

**Purpose of review:**

Recently, there has been much debate about the practicality and utility of training nephrology fellows in temporary hemodialysis catheter insertion.

**Sources of information:**

Literature review along with the authors’ opinion.

**Findings:**

This skill can be taught easily, in a controlled fashion to maximize success and minimize complications. In order to achieve this training centres should be required to teach using simulation based mastery learning and ultrasound guidance. Employing these strategies makes the inexperienced operator perform at the level of an experienced operator. As a specialty, nephrologists have a responsibility to provide hemodialysis in a timely fashion during emergencies, meaning nephrologists should be able to insert temporary hemodialysis catheters. We should take ownership over this skill and depend on no other specialty.

**Limitations:**

Limited data has been published on this subject.

**Implications:**

Temporary hemodialysis catheter insertion should be maintained as a core competency by the Royal College.

## Abrégé

**But de la revue:**

L’intérêt et l’utilité de former les *fellows* en néphrologie à l’insertion de cathéters temporaires pour hémodialyse ont récemment fait l’objet d’un débat soutenu.

**Sources d’information:**

Revue de la littérature et opinion de l’auteur.

**Résultats:**

L’insertion peut s’enseigner facilement sous supervision afin de maximiser les chances de succès et minimiser les risques de complications. Les milieux de formation devraient compléter cet enseignement par des exercices de simulation et de l’échoguidage, afin d’atteindre cet objectif. En effet, l’utilisation de ces stratégies permet à la personne en formation d’atteindre les mêmes performances que ceux qui ont de l’expérience. En tant que spécialistes, les néphrologues sont responsables, entre autres, de fournir des traitements d’hémodialyses dans des délais appropriés, et lors d’urgences. Ceci implique que les néphrologues devraient être capables d’insérer des cathéters temporaires pour hémodialyse. Nous devions nous approprier cette compétence et ne pas la déléguer à une autre spécialité médicale.

**Limites de l’étude:**

Peu de données ont été publiées sur ce sujet.

**Conséquences de l’étude:**

L’insertion de cathéters temporaires pour hémodialyse devrait demeurer une des compétences principales exigées par le Collège Royal.

## What was known before

In the nephrology community, opinions have been generated for and against the continued requirement for fellows to learn temporary hemodialysis catheter insertion. However, there has been little review of the literature to determine if this skill that can easily be taught in training.

## What this adds

The data supports the ease with which temporary hemodialysis catheter insertion can be taught when using simulation based mastery learning and ultrasound guidance. This skill should be maintained as a core competency.

Urgent dialysis catheter insertion is a life-saving treatment option in specific clinical scenarios (such as overdoses, plasma exchange, acute kidney injury etc.). Recently, there has been much debate about the practicality and utility of training nephrology fellows in temporary (non-tunneled) hemodialysis catheter insertion. Currently, the Royal College (of Physicians and Surgeons of Canada) lists this as a core competency. The position presented in this paper argues that adopting ultrasound guidance and simulation-based teaching methods results in good outcomes which can be achieved quickly (i.e. with few catheter insertions). This skill can be taught easily, in a controlled fashion to maximize success and minimize complications and should, therefore, be maintained as a core competency by the Royal College.

This paper will solely address issues around temporary hemodialysis catheter insertion. Tunneled hemodialysis catheters (a.k.a perm caths or permanent catheters) will not be addressed. The terms inexperienced and experienced operator are used throughout this discussion. The operator is the person performing the procedure. There is no uniform definition of “experience” in the studies cited. It can be assumed that inexperienced operators have inserted fewer than 25 catheters.

## What is the impact of ultrasound on catheter insertion?

The American Society of Diagnostic and Interventional Nephrology states that operators should perform 25 catheter placements to achieve competency in temporary hemodialysis catheter insertion [1]. This number is likely based on studies that compared inexperienced to experienced operators. These studies consistently found that inexperienced operators had higher complication rates [2]. Complications were mainly related to arterial puncture, hematoma and pneumothorax. However, with the advent of ultrasound guided catheter insertion, these complications have been significantly mitigated.

Thus, multiple international societies now suggest that catheter insertion should be performed using ultrasound guidance (USG). This recommendation is based on multiple randomized control trials that have demonstrated a significantly reduced complication rate and improved success rate when using real-time ultrasound guidance [3,4].

Given that ultrasound-guided insertion is the standard of care, a new question arises: What impact does USG have on catheter insertion by inexperience and experienced operator? Does USG dramatically improve the inexperienced operator? Does USG have any impact on the experienced operator? Although no randomized trials have addressed these questions specifically, data from previous studies which assessed outcomes for experienced and inexperienced operators can help inform the debate.

Prabhu et al. completed a randomized trial of 110 insertions comparing the landmark strategy to USG for femoral dialysis catheter insertion [5]. Compared with experienced operators using the landmark strategy, inexperienced operators had a much lower success rate (76 vs 100%) and two times as many complications (20% vs 10%). Whereas, when using ultrasound, the success rate (98% vs 100%) and the complication rate (5% vs 8%) were similar between inexperienced and experienced operators.

In a prospective observational study of 107 insertions, Geddes et al. compared insertion of internal jugular dialysis catheters using USG in inexperienced versus experienced operators [6]. They demonstrated that the success rate (96% vs 97%) and complication rate (0% vs 0%) was similar when comparing inexperienced and experienced operators.

Overall, the results suggest that USG has a dramatic impact on the success and complication rates for the inexperienced operator. While for the experienced operator there is a mild improvement. Most importantly, when using USG the success and complication rates are virtually identical for the inexperienced and experienced operator. There are other studies of central line insertions which have found similar results [7,8].

Ultrasound guidance is the equalizer. There will never be a randomized trial to compare inexperienced operators to experienced operators, therefore one can only use these post-hoc analyses of RCTs or observational studies to guide decision making. And the evidence is clear: USG allows the inexperienced operator to perform at the level of an experienced operator.

## How should catheter insertion be taught?

A 2012 survey found that one third of Canadian nephrology fellows felt they were not competent to insert temporary femoral or internal jugular hemodialysis catheters [9]. Similarly in a 2009 survey of recent American nephrology graduates, only two thirds of respondents felt competent to insert internal jugular hemodialysis catheters [10]. When using competency checklists for catheter insertion, many recently graduated fellows performed poorly in simulated environments. In fact, Barsuk et al. found that only 17% of traditionally trained graduates achieve a passing score on a catheter insertion checklist [11]. Clark et al., found that 0/17 trainees attained a passing score, despite most trainees having previously inserted catheters [12].

There is however good evidence to show that Simulation Based Mastery Learning (SBML) is an excellent way of training residents and fellows on how to insert catheters appropriately. SBML involves a hands-on learning environment and is highly realistic. The trainees are tested pre and post training, and must achieve a minimum passing score in order to be certified as competent. Their score is assessed using a checklist to minimize subjective grading. The simulation exercise lasts for just a few hours. At training centres where simulation modules already exist, adopting this teaching strategy requires very little resource. SBML has been shown in a variety of settings to be a more effective teaching strategy than traditional methods [13]. Clark et al., recently demonstrated that running a SBML course at the Canadian Society of Nephrology Annual Scientific Meeting resulted in a significant number of trainees achieving a passing score (from pre training 0/17 passed, post training 17/17 passed) [12].

The evidence around the impact of SBML on dialysis catheter insertion is lacking. However, the impact in the critical care literature is astounding. It has been demonstrated that SBML results in significantly reduced mechanical complications compared to traditional training. Barsuk et al. demonstrated a significant reduction in arterial punctures (14% vs. 1%) and a reduced need for catheter adjustment for SBML trainees compared to traditional teaching methods [14]. They also demonstrated a significant improvement in success rate (81% vs 95%) [14]. The implementation of an SBML program resulted in a significant 85% reduction in catheter-related bacteremias (0.50 infections per 1000 catheter-days vs 3.20 per 1000 catheter-days, P = .001) when compared to a historical control [15]. As a result of this reduction in infections it is suggested that SBML programs are extremely cost-effective [16].

## How many catheter insertions are needed for competence?

In a Canada-wide survey of nephrology fellows, Clark et al. found that the fellows inserted a median of five hemodialysis catheters six months [9]. Conservatively, it could be estimated that nephrology fellows would be inserting between 15 and 20 catheters during a two-year fellowship. Is this enough to be trained?

Maizel et al. assessed the success and complication rates of residents for temporary central line insertion [17]. The study residents had virtually no prior central line insertion experience. In total 172 insertions were performed, almost half of the lines inserted were dialysis catheters. Amongst the residents that used USG for insertion, it was found that after just 10 catheter insertions, the success rate of the residents improved to 90%. And after just four catheter insertions the complication rate plateaued at around 8%. These success and complication rates rival those of experienced operators, demonstrating that it takes fewer than 10 insertions for an inexperienced operator to become competent.

The idea that fewer catheter insertions are required for competence is reflected in other areas of medicine. For example in the 2007 guidelines, *Ultrasound-Guided Internal Jugular Access: A Proposed Standardized Approach and Implications for Training and Practice*, Dr. Feller-Kopman stated the following: [18]

“Given the rapid learning curve for US guidance of CVCs…, I would suggest… approximately 2 h of didactics, 2 h of laboratory training, and 5 to 10 proctored examinations”.

It is worth considering that Canadian fellows who are starting nephrology fellowships are not catheter naïve. They should have inserted many central lines prior to starting their fellowship (for example in their ICU and CCU rotations). Therefore, at 15 to 20 catheters during fellowship, nephrology trainees in Canada would have more than enough experience to be certified as competent.

## Other considerations

Twenty percent of Canadian nephrology fellows in the aforementioned Clark et al. survey performed no catheter insertions over a six month time period [9]. It should not be believed that this represents a lack of opportunity for catheter insertions, as there are many temporary insertions happening at teaching centres. For example, in Ontario in 2013, at the four teaching centres (Hamilton, Kingston, London, and Ottawa), there were over 650 temporary hemodialysis catheter insertions (Ontario Renal Network, unpublished data). It could be that lack of catheter insertions by fellows represents a decreased emphasis and willingness to teach this skill. Consultants in academic centres need to take an increased leadership role here. The lack of “opportunity” should not be considered a valid reason for removing this skill as training core competency.

Nephrologist should not be dependent on other services such as interventional radiology (IR) for catheter insertion for a number of reasons: First, at most centers, the IR service is not always available, which is problematic if dialysis is needed urgently (e.g. overdose, hyperkalemia). Second, it is possible that IR would not prioritize temporary hemodialysis catheter insertion, resulting in longer wait times or hospitalizations for patients waiting for catheter insertions. Third, there may be reduced complications for insertions performed by nephrologists. Significantly longer wait times, hospitalization times and worse safety outcomes have been noted for paracentesis when performed by IR versus medicine [19].

Nephrologists do not want to develop a culture of dependency as this could cripple our ability to provide urgent dialysis, a life saving therapy. If the Royal College was to remove this as a requirement of training, then which service would take ownership over temporary hemodialysis catheters insertions? In order to ensure hemodialysis remains under the sole purvey of nephrology, nephrologists must be able to provide dialysis in an emergency setting. Nephrologists cannot and should not leave temporary catheter insertions in the hands of any other service.

The data demonstrates that if an individual is trained appropriately using SBML and USG, they will have minimal difficulty inserting catheters. This is important for two reasons. First, in the authors experience, once you have been trained appropriately you will maintain an appropriate level of comfort and competence in inserting catheters even if you insert catheters only once every few years. There is no increased risk to the patient, because USG helps mitigate against complications. Further, nephrologists with a low frequency of insertion could maintain their competence by occasionally attending an SBML training program, if needed.

Second, for even those trainees who are not “procedurally oriented”, the use of SBML and USG levels the playing field (all trainees who attended Clark et al’s training achieved the passing score [12]). Catheter insertion no longer becomes a procedure which is done by “feel”. Rather, it becomes a technical skill which can easily be mastered. Therefore, even the weakest trainees can be certified as competent operators. However, it would be necessary for all nephrology training programs to have access or provide access to SBML training environments and have an ultrasound available for all insertions.

Nephrology fellowship programs should not be focused solely on training people who are destined for academia. Instead, they should prepare fellows to practice anywhere in Canada. This means that fellows must be competent in temporary dialysis catheter insertion. A trainee’s career path and centre where they will practice is never a certainty and may even change mid-career, it is essential that they learn this skill to maintain their marketability. Given the poor job prospects in nephrology in Canada, no fellow should be closing doors.

## Conclusion

The training of insertion of temporary hemodialysis catheters should be responsibility of nephrology fellowship programs. Training centres should be required to teach using SBML and USG. These techniques allow for excellent success rates and low complication rates, even after a minimal number of catheter insertions. Once trained appropriately, any nephrologist will feel comfortable with catheter insertion, even if it is years between insertions. As a specialty we have a responsibility to our patients to provide hemodialysis in a timely fashion during emergencies, meaning nephrologists should be able to insert temporary hemodialysis catheters. We should take ownership over this skill and depend on no other specialty. Temporary hemodialysis catheter insertion should be maintained as a core competency by the Royal College.

## References

[1] O’Neill WC, Ash SR, Work J, Saad TF (2008). Guidelines for training, certification, and accreditation for hemodialysis vascular access and endovascular procedures. Semin Dial.

[2] Sznajder J, Zveibil F. Central vein catheterization: failure and complication rates by three percutaneous approaches. Arch …. 1986. http://archinte.jamanetwork.com/article.aspx?articleid=606564. Accessed November 27, 2014. 10.1001/archinte.146.2.259 3947185

[3] Wu S, Ling Q, Cao L, Wang J, Xu M, Zeng W (2013). Real-time two-dimensional ultrasound guidance for central venous cannulation: a meta-analysis. Anesthesiology.

[4] Rabindranath KS, Kumar E, Shail R, Vaux E (2011). Use of real-time ultrasound guidance for the placement of hemodialysis catheters: a systematic review and meta-analysis of randomized controlled trials. Am J Kidney Dis.

[5] Prabhu MV, Juneja D, Gopal PB (2010). Ultrasound-guided femoral dialysis access placement: a single-center randomized trial. Clin J Am Soc Nephrol.

[6] Geddes CC, Walbaum D, Fox JG, Mactier RA (1998). Insertion of internal jugular temporary hemodialysis cannulae by direct ultrasound guidance–a prospective comparison of experienced and inexperienced operators. Clin Nephrol.

[7] Gualtieri E, Deppe SA, Sipperly ME, Thompson DR (1995). Subclavian venous catheterization: greater success rate for less experienced operators using ultrasound guidance. Critical care medicine.

[8] Palepu GB, Deven J, Subrahmanyam M, Mohan S (2009). Impact of ultrasonography on central venous catheter insertion in intensive care. Indian J Radiol Imaging.

[9] Clark EG, Schachter ME, Palumbo A, Knoll G, Edwards C (2013). Temporary hemodialysis catheter placement by nephrology fellows: implications for nephrology training. Am J Kidney Dis.

[10] Berns JS (2010). A survey-based evaluation of self-perceived competency after nephrology fellowship training. Clin J Am Soc Nephrol.

[11] Barsuk JH, Ahya SN, Cohen ER, McGaghie WC, Wayne DB (2009). Mastery learning of temporary hemodialysis catheter insertion by nephrology fellows using simulation technology and deliberate practice. Am J Kidney Dis.

[12] Clark EG, Paparello JJ, Wayne DB (2014). Use of a national continuing medical education meeting to provide simulation-based training in temporary hemodialysis catheter insertion skills: a pre-test post-test study. Can J Kidney Heal Dis.

[13] Cook D, Brydges R, Hamstra SJ (2012). Comparative effectiveness of technology-enhanced simulation versus other instructional methods: a systematic review and meta-analysis. Simul Healthc.

[14] Barsuk JH, McGaghie WC, Cohen ER, O’Leary KJ, Wayne DB (2009). Simulation-based mastery learning reduces complications during central venous catheter insertion in a medical intensive care unit*. Crit Care Med.

[15] Barsuk JH, Cohen ER, Feinglass J, McGaghie WC, Wayne DB (2009). Use of simulation-based education to reduce catheter-related bloodstream infections. Arch Intern Med.

[16] Cohen ER, Feinglass J, Barsuk JH (2010). Cost savings from reduced catheter-related bloodstream infection after simulation-based education for residents in a medical intensive care unit. Simul Healthc.

[17] Maizel J, Guyomarc’h L, Henon P (2014). Residents learning ultrasound-guided catheterization are not sufficiently skilled to use landmarks. Crit Care.

[18] Feller-Kopman D (2007). Ultrasound-guided internal jugular access: a proposed standardized approach and implications for training and practice. Chest.

[19] Barsuk JH, Cohen ER, Feinglass J, McGaghie WC, Wayne DB (2013). Clinical outcomes after bedside and interventional radiology paracentesis procedures. Am J Med.

